# Losing the Filter: How Kynurenine Pathway Dysregulation Impairs Habituation

**DOI:** 10.3390/cells14221786

**Published:** 2025-11-14

**Authors:** Miguel A. de la Flor, Jason C. O’Connor

**Affiliations:** 1 Department of Pharmacology, UT Health San Antonio, San Antonio, TX 78229, USA; oconnorj@uthscsa.edu; 2 Geriatric Research, Education and Clinical Center, San Antonio, TX 78229, USA; 3 Audie L. Murphy VA Hospital, South Texas Veterans Health System, San Antonio, TX 78299, USA

**Keywords:** aging, habituation, KMO, kynurenine pathway, non-associative learning and memory

## Abstract

**Highlights:**

- Kynurenine pathway (KP) dysregulation may impair habituation by disrupting synaptic plasticity, redox balance, and glial signaling.
- KP metabolites 3-HK and QA drive oxidative and excitotoxic stress, while excess KYNA interferes with neurotransmission, weakening habituation circuits.
- KMO upregulation promotes inflammatory KP activity undermining the neural flexibility required for habituation in aging.
- KMO deletion may restore KP balance and preserve olfactory and novelty habituation in aged mice.

**Abstract:**

Habituation is a fundamental form of non-associative learning that allows organisms to filter out repetitive, non-salient stimuli but declines with age. While the kynurenine pathway (KP) of tryptophan metabolism is implicated in psychiatric and neurodegenerative diseases, its role in age-related habituation deficits has been overlooked. This review proposes a systems-level framework suggesting that age-related, chronic inflammation KP dysregulation is a key driver of habituation deficits. We present evidence showing that neurotoxic metabolites from the kynurenine-3-monooxygenase (KMO)-dependent branch drive a self-reinforcing cycle of oxidative stress, excitotoxicity, and glial reactivity that destabilizes the neural circuits required for habituation. This framework redefines KP modulation as context dependent: metabolites such as kynurenic acid (KYNA), which can be disruptive when elevated in youth, may become compensatory under the oxidative load of aging. Our findings that genetic KMO deletion preserves habituation in aged and old mice provide the first direct in vivo evidence supporting this model. We propose that inhibiting the KMO branch preserves habituation not by simply altering metabolite levels but by restoring homeostatic balance across neuroimmune, redox, and plasticity networks. KMO thus emerges as a critical node for maintaining cognitive resilience, offering a therapeutic target for preserving brain function during aging.

## 1. Introduction

**Figure 1:**
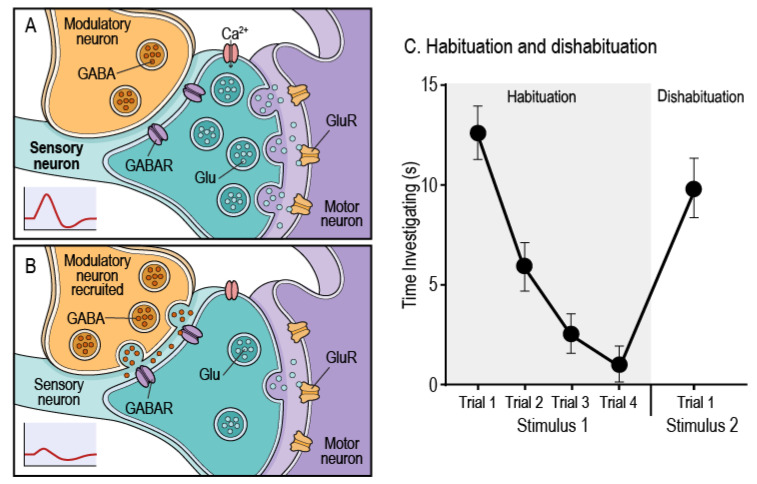
Habituation schematic and behavior. (**A**) Baseline signaling from a presynaptic sensory neuron drives glutamate release onto postsynaptic motor neuron glutamate receptors, generating strong downstream excitation, with minimal modulatory input. (**B**) With repeated stimulation, the modulatory interneuron, shown here as GABAergic, is recruited, promoting presynaptic inhibition of the sensory terminal. This reduces glutamate release and reduces postsynaptic responses, though similar modulation can also be mediated by neuromodulators such as serotonin (5-HT) or dopamine (DA). (**C**) The representative graph shows a typical habituation curve, where the time an animal investigates Stimulus 1 decreases across four trials. The subsequent presentation of a novel Stimulus 2 restores the strong investigatory response, demonstrating dishabituation.

Although often described as a simple form of learning, habituation is, in reality, a complex cognitive process that engages multiple neural mechanisms, a process that remains only partly understood [1,2]. Functionally, habituation is a form of non-associative learning characterized by a reduction in behavioral response to a repeated stimulus that is not explained by sensory adaptation or motor fatigue [3]. A key characteristic of habituation is dishabituation, where the habituated behavior is restored following the presentation of a novel or strong stimulus [4] (Figure 1C). Habituation enables an organism to ignore irrelevant, non-threatening stimuli and focus metabolically costly neural resources on salient stimuli [5,6]. By shaping how organisms respond and adapt to their environments, habituation allows higher-order cognitive functions such as selective attention, novelty detection, and associative learning paradigms like classical and operant learning to occur. Habituation is a highly conserved behavior, observed from invertebrates such as *Aplysia* and *Drosophila* to vertebrates including rodents and humans, underscoring its evolutionary adaptive importance as a fundamental mechanism for regulating neural resources [4,6]. When habituation fails, as reported in schizophrenia, autism spectrum disorder, attention-deficit/hyperactivity disorder, neurodegenerative diseases like Alzheimer’s, and in normal aging, it can lead to cognitive inflexibility and impaired learning and memory [7,8,9].

At the circuit level, habituation is rooted in coordinated synaptic strength changes, modulation of excitation-inhibition balance (E/I), and the timely recruitment of neuromodulatory systems that shape neural responses to repeated input [9,10,11] (Figure 1A,B). These molecular and cellular-level changes are expressed in both physiological and behavioral measures, including attenuated cortical evoked potentials, diminished startle responses, reduced orienting to repeated cues, and decreased investigatory behaviors [4,6,7] (Figure 1C). The consilience of evidence across these diverse mechanisms and behaviors demonstrates that habituation is an important indicator of cognitive health and function. Because the neural mechanisms underlying habituation are sensitive to metabolic states, neuroinflammatory signaling, and cellular stress responses, pathways that regulate these processes are likely to play an important role in shaping habituation across the lifespan [12,13,14].

**Figure 2:**
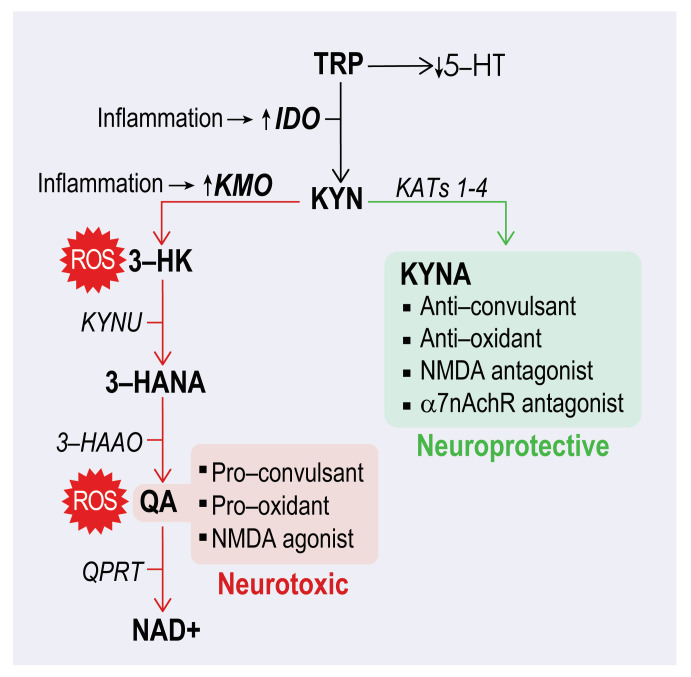
The kynurenine pathway (KP). Tryptophan (TRP) is converted to kynurenine (KYN), the central branch point of the KP. Inflammation upregulates IDO and KMO, shunting TRP into the KP and biasing it toward the KMO branch. The KMO Branch: KMO activation generates 3-hydroxykynurenine (3-HK) and quinolinic acid (QA), producing reactive oxygen species (ROS) in the process. QA is a pro-oxidant and NMDA receptor agonist. The KAT Branch: The kynurenine aminotransferases (KATs) produce kynurenic acid (KYNA), which is an antioxidant and an antagonist at NMDA and α7nAChR receptors.

Among the pathways that may impact habituation is the kynurenine pathway (KP). The KP has become a major research focus because of its central role in brain health and its links to neurological, psychiatric, and neurodegenerative diseases [14,15,16]. The KP is the primary route of tryptophan (TRP) metabolism in the central nervous system, where isoforms of indoleamine 2,3-dioxygenase (IDO) convert TRP to kynurenine (KYN). Downstream of KYN, the KP diverges into two main branches (Figure 2). The branch driven by kynurenine 3-monooxygenase (KMO) leads to the production of 3-hydroxykynurenine (3-HK) and quinolinic acid (QA), metabolites that contribute to oxidative stress and excitotoxicity, and among brain cells, KMO is mainly expressed in microglia. Ultimately the final product of the KMO branch is nicotinamide adenine dinucleotide (NAD+) a coenzyme critical for cellular energy production. The branch, mediated by kynurenine aminotransferases (KATs 1–4), which are mainly expressed in astrocytes, yields kynurenic acid (KYNA) among other metabolites. KYNA is an antagonist of NMDA and α7nACh receptors that modulate excitatory drive [17] (Figure 2). Together, these KP dynamics generate and regulate important neuroactive metabolites that influence neurotransmission, redox balance, synaptic plasticity, and neurogenesis, shaping the environment in which habituation occurs [18].

Under normal physiological conditions, the balance between the two main branches of the KP are tightly regulated [19]. However, with age-related chronic inflammation, pro-inflammatory cytokines such as IFN-γ, IL-6, and TNF-α activate the KP by upregulating IDO and KMO and channeling TRP and KYN into the KMO branch shifting the KP toward oxidative metabolism. At the same time, the reduced clearance of QA with age further skews the pathway toward excitotoxicity [20,21]. This age-related shift in KP metabolism may disrupt the conditions required for synaptic plasticity, neurotransmitter coordination, and neurogenesis, thus undermining the very processes required for habituation [22,23].

Despite substantial evidence linking KP dysregulation to increased neuroinflammation and cognitive decline, virtually all preclinical studies to date have focused on young animal models. How chronic KP activation, particularly through KMO activity, affects non-associative forms of learning such as habituation during aging remains largely unexplored. This represents a significant but tractable gap in knowledge, as aging is accompanied by chronic low-grade inflammation that upregulates IDO and KMO, shifting KP metabolism toward oxidative and excitotoxic metabolism. Our previous work and that of others has shown that KMO inhibition or deletion reduces 3-HK and QA, elevates KYNA, and attenuates oxidative and inflammatory signaling in both in vivo and in vitro models [24,25,26,27]. Interestingly, elevated KYNA has been associated with cognitive and behavioral deficits in young rodents, including schizophrenia-like phenotypes and learning impairments [28,29,30]. In contrast, our findings suggest that during aging, when excitotoxic and oxidative pressures predominate, this same shift toward higher KYNA and reduced 3-HK/QA may become neuroprotective, possibly stabilizing neural circuits rather than disrupting them. These observations imply that the functional consequences of KP modulation may be age dependent: what is maladaptive in youth may become compensatory in senescence. Together, these findings demonstrate that manipulating KMO may rebalance KP metabolism and modulate microglial reactivity. Yet whether these biochemical and neuroimmune effects extend to non-associative learning processes, particularly habituation, has not been examined systematically, an omission this review seeks to address.

Extending this framework, our recent studies provide the first empirical evidence that KMO deletion preserves habituation in aging. In our recently published work, McMullen et al. [31], old KMO-/- mice maintained robust novelty habituation in the open field, whereas age-matched WT mice did not. These findings, quantified using the coverage metric, a qualitative index of non-associative learning and exploration in the open field, suggest that KMO deletion helps preserve novelty habituation and cognitive flexibility during aging. Complementing these results, our ongoing work, de la Flor et al. [32], demonstrates that aged and old KMO-/- mice also retain olfactory habituation and dishabituation, unlike age-matched WT controls. Taken together, our studies suggest that KMO inhibition may preserve non-associative learning processes in the aging brain, implicating KP dynamics as a critical determinant of habituation function. Here we propose a new mechanistic framework that integrates existing knowledge and our recent findings to highlight a previously unrecognized role for KMO in preserving non-associative learning within the context of aging. By synthesizing evidence from multiple domains, including neurotransmitter dynamics, synaptic plasticity, oxidative stress, neuroinflammation, and adult neurogenesis, we propose how KP dysregulation may underlie age-related declines in habituation and how targeting this pathway could offer a path toward preserving cognitive flexibility during aging.

## 2. Mechanistic Effects of KP Dysregulation on Habituation

Building on this foundation, we next outline the mechanistic framework through which age-related KP dysregulation may impair habituation. This section synthesizes evidence across molecular, cellular, and circuit levels to explain how changes in KP metabolism may shape habituation during aging. Studies in invertebrate and vertebrate models demonstrate that habituation depends on the coordinated regulation of synaptic plasticity, neurotransmission, and circuit-level modulation. Under physiological conditions, repeated stimulation of neural circuits leads to activity-dependent synaptic adaptations, such as depression at excitatory synapses and enhancement of inhibitory circuit activity These adaptations are mediated by GABAergic networks and regulated by neuromodulators such as acetylcholine (Ach), dopamine (DA), and serotonin (5-HT) [2,5,7,8]. (Figure 1A,B). In the sections that follow, we examine interconnected mechanisms, including synaptic plasticity, neurotransmission, oxidative stress, neurogenesis, circuit dynamics, inflammation, and glial signaling, that together illustrate how age-related KP imbalance may undermine the neural processes underlying habituation.

We propose that reduced levels of oxidative and neurotoxic KP metabolites such as 3-HK and QA, combined with increased KYNA signaling, may help preserve non-associative learning in KMO-/- mice during aging. However, this preservation is unlikely to result solely from altered metabolite levels. In human and animal models of aging, IDO activation is often accompanied by both increases KMO and KAT activity, elevating KYNA as well as 3-HK and QA, yet cognitive impairments still emerge [20,22,27]. This highlights the importance of maintaining a balanced KP metabolic profile rather than simply favoring one branch over the other. We therefore view KMO deletion not as a unidirectional shift toward neuroprotection but as a model revealing compensatory mechanisms that may sustain neural resilience in aging. The major KP metabolites and their proposed effects on molecular mechanisms relevant to habituation are summarized in Table 1. These mechanisms may include enhanced redox buffering, altered salience or stress reactivity, persistent modulation of aryl hydrocarbon receptor (AhR)–regulated transcription, and preserved neurogenesis. Together, these adaptations may support circuit stability and efficient sensory filtering, enabling the retention of habituation capacity despite aging.

## 3. Synaptic Plasticity Impairment

**Figure 3:**
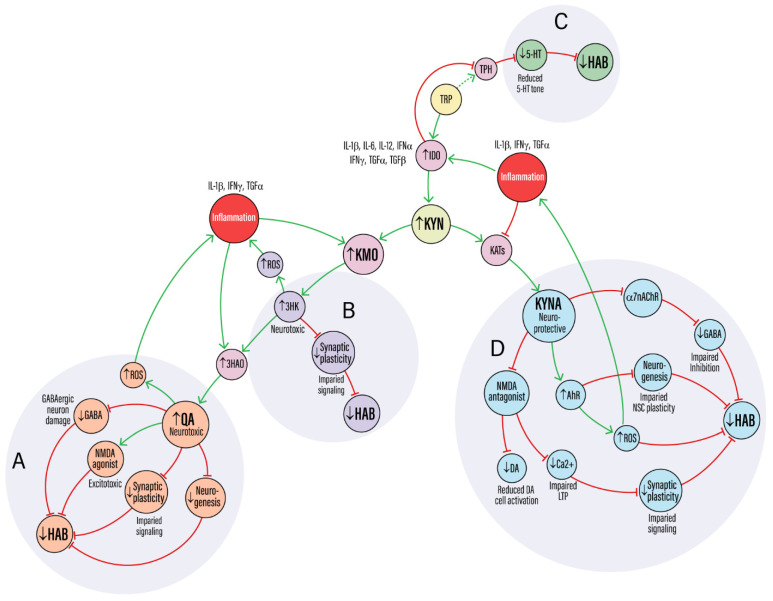
A mechanistic framework for how KP dysregulation impairs habituation. Pro-inflammatory cytokines upregulate IDO and KMO, shunting TRP toward the neurotoxic KP branch. (**A**,**B**) KMO activation generates 3-HK and QA, which drive ROS, NMDA receptor-mediated excitotoxicity, GABAergic neuron damage, and impaired neurogenesis. These insults converge to degrade synaptic plasticity and impair habituation (↓HAB). (**C**) Inflammation-driven IDO activation diverts TRP from the serotonin (5-HT) pathway, reducing 5-HT availability. This weakens a key neuromodulatory system required for sensory filtering and habituation. (**D**) The paradoxical KAT branch: In excess, KYNA becomes maladaptive. Its antagonism of NMDA and α7nAChR receptors disrupts LTP and impairs GABAergic and DA signaling. Concurrently, activation of the AhR by KYN and, to a lesser extent, KYNA, suppresses NSC plasticity, with all pathways converging to impair habituation.

Synaptic plasticity, particularly long-term potentiation (LTP) and long-term depression (LTD), enables the brain to encode stimulus salience and adapt to repeated input, providing a cellular substrate for habituation [2,7,46]. Both the neurotoxic (KMO) and neuroprotective (KAT) branches of the KP may modulate synaptic plasticity in ways that influences habituation [47,48]. KYNA’s actions on NMDA and α7nACh receptors, outlined in Table 1, directly affect synaptic efficacy and plasticity. For instance, KYNA, produced by KATs in astrocytes, is an antagonist of NMDA receptors and a regulator of α7nACh receptors. By blocking postsynaptic NMDA receptors, KYNA may limit calcium influx required for LTP induction, while its antagonism of presynaptic α7nAChRs could reduce glutamate release, jointly dampening activity-dependent synaptic plasticity (Figure 3D) [33,38,49]. These effects are most prominent in the hippocampus and prefrontal cortex, which regulate attentional flexibility, stimulus filtering, and memory encoding, all core components of habituation in vertebrate models. KYNA mediated suppression of LTP is dose dependent and reversible and LTP deficits in KMO-/- mice are reversed by KAT II inhibition, suggesting that excessive KYNA impairs synaptic efficacy [47,50].

Conversely, the KMO-dependent branch generates QA, an endogenous NMDA receptor agonist that drives calcium overload, excitotoxicity, and dendritic retraction [36] (Figure 3A). QA further exacerbates this excitotoxicity by inhibiting astrocytic glutamate uptake, leading to elevated extracellular glutamate concentrations and prolonged NMDA receptor activation [25,44]. This environment may selectively injure GABAergic interneurons, disrupting inhibitory tone and possibly collapsing the E/I balance essential for habituation (Figure 3A).

Thus, KP dysregulation may disrupt NMDA-dependent plasticity through opposing mechanisms, either excessive suppression by KYNA or overactivation by QA, both of which could lead to loss of synaptic plasticity. ROS generated by 3-HK and QA may further disrupt plasticity by degrading redox sensitive signaling proteins involved in cytoskeletal remodeling, receptor trafficking, and maintenance of perineuronal nets (Figure 3A,B). This oxidative stress may interfere with the structural changes needed to consolidate synaptic strength and prune redundant inputs, possibly undermining the adaptive filtering that supports habituation [51,52,53]. Conversely, in KMO-/- mice, reduced production of these neurotoxic metabolites may shift the redox environment toward greater ROS buffering capacity, potentially limiting oxidative damage to plasticity-related proteins [38,54]. Such protection could permit compensatory activation of CREB- and BDNF-dependent transcriptional programs that sustain dendritic integrity and activity-dependent remodeling despite elevated KYNA [55].

## 4. Neurotransmitter Disruption

KP metabolites, particularly KYNA, may significantly disrupt neurotransmitter systems critical for habituation [50,56]. KYNA’s inhibition of NMDA and α7nACh receptors, which are often located presynaptically, reduces calcium influx into neuron terminals. This leads to a decrease in presynaptic glutamate release, as calcium is essential for vesicle fusion and neurotransmitter release [33,38] (Figure 3D). This reduction in glutamatergic signaling may propagate across networks, as glutamate provides the primary excitatory drive to many GABAergic interneurons. By suppressing glutamate release, KYNA may reduce the excitatory drive on these inhibitory GABAergic interneurons, leading to a secondary suppression of GABA release [56,57]. Experimental increase in KYNA in the rodent hippocampus reduces extracellular glutamate and suppresses α7nAChR-mediated excitation of interneurons, effects reversed by KAT II inhibition [49,57]. This disinhibition may blunt the brain’s capacity to filter repetitive inputs and allocate attention, disrupting the adaptive neural filtering at the core of habituation.

Changes in glutamatergic and GABAergic transmission induced by KP dysregulation could profoundly affect dopamine (DA) signaling, which encodes stimulus novelty and salience, key mechanisms of habituation [16,58,59]. Attenuation of DA signaling during habituation involves synaptic plasticity and shifts in DA neuron firing patterns, processes modulated by glutamate and GABA. In vertebrate models, reduced glutamatergic input to the ventral tegmental area, coupled with altered GABAergic tone, impairs the burst firing of DA neurons and decreases DA release [9,59,60] (Figure 3D). Consistent with this, disruptions in KP metabolism can alter dopaminergic transmission. Rodent studies reveal that increasing KYNA levels dampens DA release in the prefrontal cortex and nucleus accumbens, likely through reduced α7nAChR signaling on dopaminergic terminals, a change reversed by KAT II inhibition [14,50,61]. Through these combined actions on glutamate, GABA, and DA systems, KYNA appears to compromise the signaling precision required for normal habituation. These mechanisms are summarized in Table 1, where altered KP metabolite levels disrupt glutamatergic, GABAergic, and dopaminergic signaling that may be essential for habituation.

Serotonin (5-HT) is a key neuromodulator that regulates habituation across species, with experimental and comparative studies showing that serotonergic signaling shapes behavioral flexibility [62,63,64]. KP activity may regulate 5-HT availability, as inflammation-induced IDO activation diverts TRP away from tryptophan hydroxylase, the rate-limiting step in 5-HT synthesis, toward KYN production, plausibly reducing 5-HT synthesis. The resulting loss of serotonergic tone may diminish sensory gating and cognitive flexibility, thereby limiting habituation function [43,45,65] (Figure 3C). In rodents, pharmacological or age-related reductions in 5-HT impair habituation to novel environments, while serotonergic activation can enhance this adaptive response [63]. Similarly, 5-HT-dependent habituation of the acoustic startle reflex in zebrafish highlights a conserved role for serotonergic signaling in regulating sensory gating and behavioral flexibility [66].

QA, a neurotoxic metabolite downstream of KMO, may also contribute significantly to habituation deficits by disrupting the balance of glutamatergic and GABAergic signaling [67] (Figure 3A). Unlike KYNA, QA acts as an NMDA receptor agonist, leading to unregulated calcium influx into neurons and triggering a cascade of excitotoxic events that culminate in dendritic degeneration and neuronal loss. In hippocampal and cortical culture models, QA exposure produces neuronal loss and synaptic degeneration, effects accompanied by lipid peroxidation and mitochondrial ROS generation [35,36,45]. QA further exacerbates these conditions by inhibiting astrocytic glutamate uptake through excitatory amino acid transporters, thereby elevating extracellular glutamate levels and prolonging NMDA receptor activation [25,37,68] (Figure 3A). The resulting excitotoxic stress and loss of GABAergic inhibition destabilize local circuits needed for filtering irrelevant sensory inputs and thus habituation. In vivo, intracerebral QA administration in rodents impairs spatial learning and attentional flexibility, suggesting that QA-induced excitotoxicity extends to behavioral domains relevant to habituation [69,70,71]. The convergence of glutamate excitotoxicity and reduced inhibitory tone thus creates an environment of neuronal hyperexcitability that may underlie habituation deficits [2,18,72]. Conversely, in KMO-/- mice, the chronic reduction in neurotoxic metabolites such as QA may favor adaptive receptor-level changes, like increased NMDA receptor subunit expression, that help stabilize E/I balance and preserve habituation during aging.

## 5. Oxidative Stress and Mitochondrial Dysfunction

The neurotoxic KP metabolites 3-HK and QA drive oxidative stress through distinct yet converging mechanisms. 3-HK undergoes auto-oxidation and redox cycling, generating superoxide and other ROS that directly damage cellular components [35,67] (Figure 3B). This redox cycling can also deplete glutathione (GSH), lowering the GSH/GSSG ratio and weakening mitochondrial antioxidant defenses such as SOD2 [73,74]. QA acts as an NMDA receptor agonist, promoting excessive calcium influx that overwhelms neuronal and mitochondrial buffering capacity and may indirectly produce calcium overload characteristic of excitotoxicity [37,70] (Figure 3A). Calcium overload may trigger opening of the mitochondrial permeability transition pore (mPTP), collapsing membrane potential, impairing oxidative phosphorylation, particularly complex I activity, and promoting cytochrome c release and bioenergetic failure. NMDA-driven activation of neuronal nitric oxide synthase (nNOS) may further elevate nitric oxide and peroxynitrite, nitrating mitochondrial proteins and amplifying respiratory dysfunction [51,74]. The combined increase in ROS and mitochondrial calcium overload are thought to contribute to oxidative stress, which together may impair mitochondrial ATP synthesis, disrupt membrane integrity, and reduce neuronal resilience to energy demands [75,76]. These deficits may compromise the synaptic plasticity and network stability required for habituation, as summarized in Table 1.

Mitochondria in prefrontal and hippocampal neurons, which are highly active during learning and memory formation, are especially vulnerable to this redox burden [77,78]. Calcium-dependent activation of the fission protein DRP1 may fragment mitochondrial networks and limit spine-localized ATP supply, while sustained oxidative stress can impair PINK1–Parkin–dependent mitophagy, allowing accumulation of damaged mitochondria. This vulnerability is exacerbated by the natural decline in mitochondrial efficiency that occurs with aging [51,75,79]. When KP-induced oxidative stress is superimposed on this decline, it may accelerate synaptic failure and reduce network adaptability. ROS and lipid peroxidation may degrade synaptic proteins and receptor scaffolds such as NMDA and AMPA receptors, and also disrupt cytoskeletal remodeling [73,74]. These structural alterations compromise dendritic spine stability and synaptic efficacy, weakening the plasticity mechanisms that may support habituation. Similar mitochondrial and redox signatures are observed in aging cortex and in neurodegenerative diseases such as Alzheimer’s and Parkinson’s, suggesting shared mechanisms of mitochondrial stress and synaptic vulnerability [51,76].

Cumulative mitochondrial damage and oxidative stress impair the synaptic machinery required for activity-dependent plasticity, reducing the brain’s ability to adapt to repeated or predictable stimuli [80]. The resulting ATP reduction and compromised protein function lead to a breakdown in signal transduction, directly impairing synaptic plasticity and the ability of neurons to maintain their structural integrity [42,51,77]. Oxidative modification of redox-sensitive signaling nodes, including CaMKII, cofilin, and NMDA receptor redox sites, may blunt LTP/LTD signaling and activity-dependent remodeling [51,78]. Consequently, the brain’s ability to register stimulus redundancy and suppress behavioral output, the core function of habituation, may become compromised. This chronic excitotoxic state and reduced metabolic support may prevent the fine-tuned synaptic adjustments needed for habituation. In KMO-/- mice, reduction in 3-HK and QA production may shift the redox baseline, reducing cumulative oxidative damage with age. Accordingly, by reducing 3-HK and QA while elevating KYNA, KMO deletion would be expected to mitigate oxidative stress within key brain regions, supporting circuit integrity and preserved habituation [35,73]. This enhanced redox buffering capacity could help preserve the synaptic plasticity required for habituation by protecting mitochondrial integrity and maintaining energy-dependent signaling mechanisms.

## 6. Neurogenesis Impairment

Adult neurogenesis is strongly shaped by KP balance, [81,82], with the relevant metabolite actions summarized in Table 1. Studies suggest that KYNA can suppress neural stem cell (NSC) proliferation in the ventricular-subventricular zone (SVZ) and dentate gyrus (DG) of the hippocampus through AhR signaling, while 3-HK and QA promote oxidative stress that impairs neuronal survival [25,41,83]. These metabolite-specific actions, mediated through transcriptional and signaling pathways, highlight the dual role of the KP in regulating neurogenesis. Disruptions to this balance, often triggered by age-related inflammation, shift the KP toward oxidative and neurotoxic metabolism, undermining neurogenesis and weakening circuit functions critical for cognitive resilience, possibly including habituation [17]. In aging, sustained low-grade inflammation activates IDO increasing activity through the KMO branch; where direct aging data are limited, we leverage injury and disease models as mechanistic frameworks rather than direct surrogates.

AhR signaling is a key regulator of NSC proliferation and differentiation [41,82,83,84] (Figure 3D). In adult zebrafish models of Alzheimer’s disease, amyloid toxicity reduces KAT 2 expression, leading to lower KYNA levels. Because KYNA normally suppresses NSC proliferation through AhR signaling, this reduction removes that inhibition and allows a regenerative neurogenic response [40,82]. Similarly, in a mouse model of stroke, inhibiting AhR increased NSC proliferation in the ventricular-subventricular zone and the subgranular zone of the hippocampus and upregulated of pro-neurogenic transcription factors such as Neurogenin 1 and Neurogenin 2 [83]. These findings suggest the directionality of AhR effects on proliferation and provide a framework for testing hypotheses in the context of aging, where homeostatic neurogenesis declines more gradually. Mechanistically, AhR activation can induce expression of the cell-cycle inhibitor p21 and reduce Cyclin D1 [85,86]. Conversely, reduced AhR signaling may permit pro-neurogenic programs (e.g., Wnt/Notch) in some settings, though the relevance of this effect during normal aging is not yet established [82,83,84] (Figure 3D). Because adult-born neurons in the DG and SVZ contribute to circuit plasticity, changes in AhR-driven neurogenesis may directly influence habituation, which depends on the integration of new neurons into these networks.

Excessive production of 3-HK and QA skews the KP toward neurotoxicity, creating a hostile neural environment. QA, in particular, acts as excitotoxin, overstimulating NMDA receptors and driving calcium overload that fuels ROS generation and cellular injury [14,36,37] (Figure 3A,B). This may lead to neuronal loss, disrupt cytoskeletal architecture, and promote a self-perpetuating cycle of neuroinflammation [44,74]. By degrading synaptic integrity these processes may weaken the circuit dynamics required for habituation. Moreover, the presence of QA creates an unfavorable environment for NSC survival and proliferation, limiting the neurogenic plasticity that supports habituation.

Other evidence points to a pro-neurogenic role for the KP, where its metabolites help maintain stem cell identity and regenerative potential. In human embryonic stem cells (hESC) and induced pluripotent stem cells (iPSCs), endogenous KYN production activates AhR, which promotes the expression of self-renewal genes like *POU5F1* and *NANOG*. This KYN-AhR signaling loop is further reinforced by feedback activation of IDO1 and AhR, stabilizing the pluripotent state [40,41,87]. For differentiation to occur, this signaling must be attenuated. During ectodermal lineage commitment, KYN levels decrease through activation of KAT2, which catalyzes the conversion of KYN to KYNA, a metabolic shift essential for exit from pluripotency. While hESCs and iPSCs are distinct from adult NSCs, these findings provide useful mechanistic analogies, illustrating how KYN- and KYNA-mediated AhR signaling may regulate cell fate programs relevant to habituation [41,82].

KP metabolites act as context-dependent regulators of neurogenesis. During inflammation, they inhibit stem cell plasticity, whereas under basal conditions, AhR-mediated signaling may support pluripotency [41,83]. Understanding these interactions is crucial for developing therapeutic strategies that can modulate the KP to promote brain repair. Our observation of increased DCX and EdU labeling in the SVZ of old KMO-/- mice provides direct in vivo evidence that reducing chronic exposure to 3-HK and QA preserves the neurogenic niche from age-related decline [32]. Whether KMO deletion actively biases NSCs toward pro-neurogenic fates remains a key question for future experiments. Together, these mechanisms plausibly help maintain circuit function in regions such as the hippocampus and olfactory bulb, thereby supporting olfactory and novelty-related habituation.

## 7. Circuit-Level Disruption

KP dysregulation may impair circuit-level coordination across the brain, disrupting the dynamic interactions that support habituation. This effect is most evident in regions such as the hippocampus, prefrontal cortex, and sensory cortices, which are highly sensitive to E/I imbalance and timing-dependent plasticity [56,88]. KYNA reduces both excitatory and inhibitory tone as discussed previously, but may disproportionately suppress inhibitory transmission by blocking α7nAChRs on GABAergic terminals [14,30] (Figure 3D). QA increases the susceptibility of GABAergic medium spiny neurons in the striatum to excitotoxic and oxidative stress, leading to weakened inhibition and disorganized network activity [36,61] (Figure 3A). The resulting loss of inhibitory signaling destabilizes E/I balance, eroding the signal-to-noise ratio that enables the brain to filter redundant stimuli and engage in habituation. Because the olfactory bulb and hippocampus rely on similar PV+ interneurons and gamma-band dynamics, it is plausible that these same KP-driven mechanisms extend to circuits underlying olfactory and novelty habituation [56,72,89].

In the hippocampus and olfactory bulb, disruptions in KP metabolism may interfere with rhythmic coordination and sensory gating, undermining the circuit dynamics required for habituation [56,90]. Altered theta and gamma oscillations can compromise the brain’s ability to register stimulus redundancy and selectively suppress behavioral responses [89,91]. Consistent with this, changes in KYNA and QA levels have been linked to reduced evoked potential habituation, prepulse inhibition, and behavioral responses, suggesting that KP imbalance can directly influence habituation-related processes [50,57,88]. Our data indicate that KMO-/- mice retain olfactory and novelty habituation into old age [31,32], implying that the absence of chronic oxidative KP activity may protect inhibitory circuits. By limiting the production of neurotoxic 3-HK and QA, KMO deletion may preserve interneurons, maintain inhibitory tone, and sustain oscillatory coherence in the hippocampus and olfactory bulb, mechanisms that together support circuit stability and cognitive resilience during aging.

## 8. Inflammation-Driven Feedback Loops

Neuroinflammation and KP activation reinforce one another, forming a self-amplifying cycle that intensifies neuronal stress. Pro-inflammatory cytokines such as IFNγ, IL-6, and TNFα upregulate IDO and KMO, diverting KYN toward the neurotoxic branch and sustaining production of 3-HK and QA [16,17,73]. These metabolites, in turn, amplify oxidative stress, disrupt redox signaling, and activate microglia, creating a self-reinforcing positive feedback loop of inflammation, synaptic dysfunction, and neuronal loss [67,68]. For example, 3-HK drives ROS production that feeds back into cytokine signaling (Figure 3B), while QA overstimulates NMDA receptors and promotes calcium-dependent ROS generation, and under certain conditions KYNA can also contribute to mitochondrial oxidative stress [18,35]. Prolonged inflammation further biases the KP toward neurotoxic dominance by selectively suppressing KAT activity and reducing KYNA production, thereby intensifying oxidative stress and weakening circuit stability (Figure 3D).

These inflammatory-KP feedback loops are implicated across neurodegenerative conditions and may become entrenched during aging, progressively undermining the circuits that support habituation [27]. In disease models such as Huntington’s, KMO deletion reduces peripheral cytokine levels and microglial reactivity, even in the absence of full behavioral rescue [54]. These findings suggest that KP metabolites are not passive byproducts of inflammation but active modulators of neuroimmune tone.

In KMO-/- mice, the reduction in chronic oxidative and neurotoxic activity may dampen microglial activation, consistent with our previous findings that pharmacological inhibition of KMO blunts LPS-induced activation in BV2 microglia and that primary microglia from KMO-/- mice show a reduced pro-inflammatory response to LPS [24]. This attenuation of microglial reactivity may interrupt degenerative feedback loops, lower basal cytokine tone, and protect vulnerable circuits from inflammatory stress. This immune-metabolic insulation could preserve the synaptic plasticity necessary for habituation, particularly under age-related inflammatory load.

## 9. AhR Activation and Glial Crosstalk

Through signaling by KYN, and to a lesser extent KYNA, AhR functions as a central hub of glial crosstalk that regulates oxidative stress responses, immune tone, and neurogenesis [13,82] (Figure 3D). AhR is broadly expressed in astrocytes, microglia, and NSCs, positioning it as a key integrator of glial communication, metabolic state, and experience-dependent plasticity [83]. Under inflammatory conditions, KYN-mediated AhR activation induces IDO1 and SLC7A5, reinforcing KP activity and sustaining a feedback loop of inflammation and oxidative stress [13,27]. This cascade can disrupt glial signaling and weaken circuit stability, potentially compromising the neural adaptation processes that underlie habituation.

Persistent AhR signaling weakens antioxidant defenses and downregulates neurotrophic and plasticity-related genes, undermining synaptic remodeling and NSC maintenance that support adaptive processes like habituation [83] (Figure 3D). As a result, chronic AhR activation contributes to a shift in glial phenotype toward a reactive, pro-inflammatory phenotype, while diminishing regenerative potential in aging circuits [27,83]. By pushing glia into sustained reactive, pro-inflammatory states, chronic AhR activation may compound mitochondrial stress and synaptic instability, progressively undermining habituation-related circuits.

AhR activity is highly ligand- and context-specific, producing either neurotoxic or neuroprotective outcomes depending on the stimulus [13,83]. Similarly, KYNA acts as a weak AhR agonist, which under non-inflammatory conditions may bias signaling toward homeostatic or antioxidant programs rather than pro-inflammatory ones [27]. This duality underscores the limits of interpreting KYNA’s effects solely through AhR activation and cautions against viewing KYNA as uniformly protective or KMO activity as entirely detrimental.

In KMO-/- mice, reduced KYN-driven activation of the KP–AhR pathway may lessen chronic engagement of stress-related transcriptional programs, even though KYNA levels remain modestly elevated. This restraint may preserve glial homeostasis, antioxidant capacity, and neurotrophic signaling, factors essential the neural plasticity and circuit coherence required for habituation in aging. By linking metabolic state, inflammatory tone, and environmental cues, AhR emerges as a molecular bridge between KP dynamics and long-term cognitive flexibility, including adaptive processes such as habituation.

## 10. Summary

This review proposes a mechanistic framework linking age-related KP dysregulation to deficits in habituation, integrating evidence across molecular, cellular, and circuit levels. While KP metabolites have been extensively studied in contexts such as inflammation, immunomodulation, and neurodegenerative disease, their direct impact on non-associative learning has been largely overlooked. Habituation, as a fundamental form of learning and memory, underpins higher-order cognitive processes such as attention, associative learning and memory, and decision-making. Because habituation deficits are pervasive across neuropsychiatric and neurodegenerative disorders, understanding how KP balance may impact habituation may provide novel insights into cognitive resilience and decline in aging.

The mechanisms outlined here collectively demonstrate that age-related KP dysregulation may destabilize neural adaptation through convergent effects on synaptic plasticity, neurotransmitter signaling, oxidative balance, neurogenesis, network stability, and glial communication. Neurotoxic metabolites such as 3-HK and QA drive oxidative stress, excitotoxicity, and inhibitory signaling failure, while inflammation amplifies these effects through cytokine–KP feedback loops. Limiting KMO activity may restore equilibrium across these interdependent systems, stabilizing redox tone, preserving inhibitory circuitry, and dampening neuroinflammatory drive. Evidence from our work with KMO inhibition and the broader literature shows that KMO inhibition reduces the neurotoxic KP metabolites downstream of KMO and has detectable effects on inflammation and microglia reactivity. Consistent with previous reports, we have reported that both pharmacological inhibition and genetic deletion of KMO attenuate microglial activation and inflammatory signaling. In BV2 microglia and primary cultures from KMO-/- mice, KMO inhibition blunted LPS-induced cytokine responses, suggesting that reduced activity through the KMO branch of the KP limits oxidative and excitotoxic stress [24]. Consistent with these cellular effects, we recently reported that old KMO-/- mice maintain robust novelty habituation in the open field but not age-matched WT controls [31]. This suggests KMO deletion may confer neuroprotection during aging in mice. Our ongoing work demonstrates that aged and old KMO-/- mice maintain olfactory habituation and dishabituation indicating that this KP metabolic rebalancing supports the preservation of non-associative learning into old age [32].

Within the context of habituation these findings redefine KP modulation as an important regulator of neural homeostasis rather than a simple shift between “toxic” and “protective” metabolism. Elevated KYNA, while potentially disruptive in youth, may become compensatory under aging-related oxidative load, mitigating excitotoxic and inflammatory stress. Together, these insights define a systems-level model in which age-related KP imbalance erodes habituation by coupling oxidative, inflammatory, and neuroplastic stress into a self-reinforcing cycle. Thus, inhibiting KMO may lessen oxidative, neurotoxic, and excitotoxic stress enough to preserve the neural stability required for habituation during aging. While studies with global KMO-/- mice provide valuable insights, as reported here, they cannot distinguish the relative contribution of specific cell types or developmental timing to the observed behavioral effects. Because KMO is expressed in most cell types, constitutive deletion may produce compensatory changes that confound interpretation. Future work should therefore employ spatially and temporally inducible systems to inhibit KMO in defined neural populations or at specific life stages, allowing direct testing of when and where KMO activity most strongly impacts habituation. In parallel, targeting 3-hydroxyanthranilic acid oxidase (3-HAO), the enzyme downstream of KMO could clarify how downstream metabolic activity contributes to oxidative stress and behavioral outcomes. These approaches would refine our mechanistic understanding of how selective modulation of the KP shapes non-associative learning during aging.

## Figures and Tables

**Table 1:** Effects of major KP metabolites on mechanisms underlying habituation.

Metabolite	Mechanisms Affected	Key References	Impact on Habituation
Kynurenic acid (KYNA)	Antagonist at NMDA and α7nACh receptors; suppresses glutamate release; reduces excitatory drive to GABAergic interneurons; activates AhR signaling	[14,17,18,33]	Dose-dependent suppression of LTP; disrupted glutamate–GABA balance; reduced DA signaling; excessive AhR activation reduces neurogenesis and circuit stability → impairs habituation
3-Hydroxykynurenine (3-HK)	Undergoes auto-oxidation and redox cycling, generating ROS; contributes to oxidative stress and lipid peroxidation	[16,25,34,35]	Oxidative damage to redox-sensitive synaptic proteins and cytoskeletal elements; impaired mitochondrial function and synaptic plasticity → impairs habituation
Quinolinic acid (QA)	NMDA receptor agonist; drives calcium overload and excitotoxicity; inhibits glutamate uptake; induces cytotoxicity in GABAergic neurons	[36,37,38]	Excitotoxicity and loss of inhibitory tone destabilize circuits; suppresses neurogenesis; chronic E/I imbalance → strongly impairs habituation
Kynurenine (KYN)	Precursor metabolite; activates AhR signaling to maintain stem-cell identity and pluripotency; modulates immune tone	[17,39,40,41]	Context-dependent: supports regenerative potential under basal conditions but may suppress differentiation and neurogenesis during aging/inflammation → mixed effects on habituation
↑ IDO activity (↓ 5-HT)	Inflammation-driven IDO activation diverts tryptophan away from serotonin (5-HT) synthesis	[42,43,44,45]	Decreased 5-HT availability impairs sensory gating, salience detection, and cognitive flexibility → impairs habituation

## Data Availability

Not applicable.
